# Determination of characterization, antibacterial and drug release properties of POSS-based film synthesized with sol-gel technique

**DOI:** 10.3906/kim-2101-56

**Published:** 2021-06-30

**Authors:** İdil KARACA AÇARI, Süleyman KÖYTEPE, Burhan ATEŞ, İsmet YILMAZ, Turgay SEÇKİN

**Affiliations:** 1 Department of Bioengineering, Faculty of Engineering and Natural Sciences, Malatya Turgut Özal University, Malatya, Turkey; 2 Department of Chemistry, Faculty of Science, İnönü University, Malatya, Turkey

**Keywords:** Polyhedral oligomeric silsesquioxane, dip coating, antibacterial activity, drug delivery

## Abstract

In the study, antibacterial film synthesis was aimed using sol-gel technique from POSS structure with various functional groups. For this purpose, antibacterial properties have been acquired by metronidazole to the films to be synthesized. The films obtained were coated on glass surface samples by dip coating method. Antibacterial activities of surface coated glass samples were observed in *E.coli* and *S. aureus* bacteria. Metronidazole release studies in the film samples were followed by UV spectrophotometer. It was observed that drug release reached 68.90% at the end of the 24th h. As a result, it is thought that the synthesized film will be a good candidate especially for biomedical surface coating areas.

## 1. Introduction

Polyhedral oligomeric silsesquioxane (POSS), which belongs to the silsesquioxane family, was first synthesized in 1946. It has a 50 A° cage width and shows rhombohedral crystal braid [1,2]. The POSS molecule has a three-dimensional cubic lattice structure and this cubic lattice is represented by the closed formula (RSiO_1.5_)_n_. These R groups in the POSS structure may be amine, halogen, mercapto, glycidyl, vinyl functional alkyl, alkylene or aromatic groups, or only hydrogen atoms [3–11]. POSS structures can easily be functionalized by attaching different groups to the eight corners of the cubic structure of POSS [12,13]. The fact that POSS can be functional makes the material very attractive. POSS structures, with properties such as biocompatibility, and chemical and thermal stability, are becoming high performance nanotechnologies for medical, aerospace, mechanical and optoelectronic applications [14,15]. Nitro-containing imidazoles have significant antimicrobial activity. Those such as benznidazole, secidazole, metronidazole and ornidazole are used clinically [16–18].

In this study, POSS-based film coating material was synthesized by the sol-gel method. The synthesis was confirmed by commonly used characterization methods. Characterization and surface morphology-mapping were carried out for the synthesized films by Fourier transform infrared spectrometer (FTIR), scanning electron microscope-energy dispersive X-Ray (SEM-EDX), atomic force microscopy (AFM) and upright optic microscope techniques. In addition, liquid (water) contact angles were determined by an automatic contact angle analyzer. Antibacterial activity was determined by the broth microdilution method, which is quite common. Results are given as the minimum inhibitory concentration (MIC) values. Metronidazole release was monitored at 340 nm with a UV-Spectrophotometer.

## 2. Experimental

### 2.1. Chemicals and devices

Chemicals and solvents used in the synthesis and coating steps were obtained from Sigma-Aldrich. Escherichia coli (*E.coli*) ATCC: 25922 and Staphylococcus aureus (*S. aureus*) ATCC: 29213 bacteria, used to determine the antibacterial properties of the prepared coatings, obtained from the medical faculty of İnönü University. Müller Hinton Agar and Müller Hinton Broth (Or-Bak) were purchased Merck. The instruments used in the characterization of the structures were listed here. Infrared spectrum of the prepared antibacterial coatings was recorded with in the range 4000–400 cm^−1^ on a Perkin Elmer Spectrum Two model Fourier Transform Infrared Spectrometer. Leo EV40 brand SEM device was used. In addition to high-resolution images taken at different magnifications, EDX (Energy Distribution X-rays) analysis was performed. A type of mapping was made by taking EDX analysis from the surface where the homogeneity on the sample surface was captured. Surface morphology was also determined in the noncontact mode with the Atomic Force Microscopy (AFM, XE-100E; Park Systems Corp.) device and upright optic microscope (M-Shot Metallurgical Microscopes). Water contact angle measurement of the glass surface (GS) and POSS-metronidazole coated GS surface were obtained using water drop technique by SEO – Phoenix 300 Touch Automatic Contact Angle Analyser.

### 2.2. Synthesis of the POSS-based film coating structure with sol-gel method

**Figure 1:**
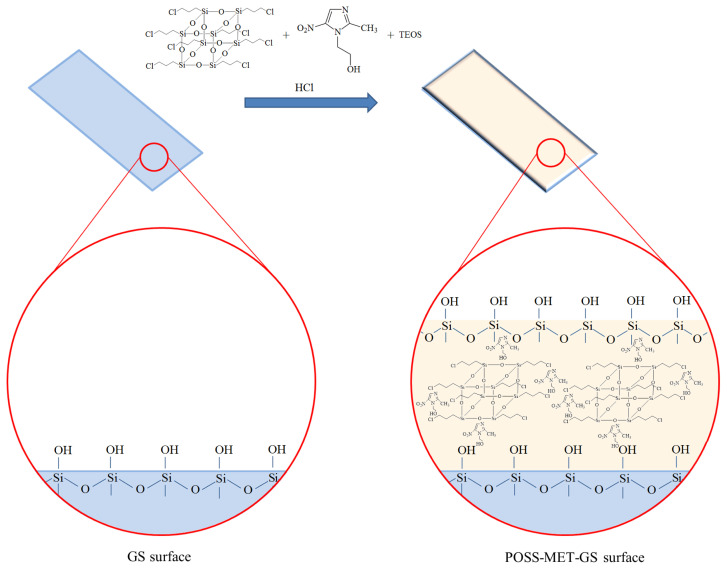
Scheme showing the synthesis of POSS film structures containing metronidazole.

The chloro-functional POSS (0.1 g), metronidazole (0.8 g), tetraethyll orthosilicate (TEOS) (10 mL) and HCl (as catalyst, 1M 1mL) were mixed by ultrasonic agitation for 1h and then magnetically stirred for 24 h at room temperature [19]. The acidic sol mixture was obtained (Figure 1). The metronidazole containing POSS film coating structure, which is synthesized with the sol-gel technique, will give its antibacterial property through the metronidazole in its structure.

### 2.3. Coating glass surfaces with POSS-based film

Firstly, glass surfaces were cleaned before coating to ensure a homogeneous coating. Mechanical washing in detergent water, rising in deionized water, dissolving in ammonia water, rinsing in deionized water, rinsing in acetone and drying operations were performed. Prepared sol was coated on glass surface with dip coating method [20]. Sol-gel dip coating is accomplished by drawing a substrate from a metal alkoxide solution. In this process, a homogeneously solid film accumulated on the substrate surface with drainage by gravity, solvent evaporation and condensation reactions [21]. According to the classical dip coating method the glass surfaces cleaned were dipped into the prepared sol solution for 2 min and withdrawn at a certain speed. This procedure was repeated 5 times. The coated glass surfaces were then allowed to dry at room temperature for 24 h.

### 2.4. Drug release for metronidazole

UV spectrophotometric measurement was used to determine metronidazole release potential. The maximum absorbance value of metronidazole was observed at 340 nm. Metronidazole calibration graph was made. The samples were allowed to stand at 37°C in phosphate buffered saline (PBS, pH = 7.4) [22]. Absorbance values were read at the 10, 20, 30, 40, 50th min and 1, 2, 3, 4, 5, 6, 12th and 24th h. The absorbance of the samples taken from release media at certain time invervals were measured in a UV spectrophotometer at 340 nm and metronidazole concentrations were calculated from the calibration graph curve given. The percentage drug release was calculated at each sampling time.

### 2.5. Antibacterial activity of POSS-based film coating structure

The broth microdilution method, quantitative reference method routinely used in clinical laboratories, was used for determine the minimum inhibitory concentrations (MICs) of *E.coli* (ATCC 25922) and *S.aureus* (ATCC 29213) [23]. Initially the bacterial suspension was prepared according to 0.5 McFarland. Mueller Hinton Broth (MHB), sample and bacterial suspension was placed in each well in sterile 96-well plates. The last well was selected as the control. Incubate at 37 °C for 24 h. Samples were incubated on Mueller Hinton Agar medium after incubation. After culture, the petri dishes were incubated at 37 °C for 24 h. Then the minimum inhibitory concentration (MIC) values were determined.

## 3. Results and discussion

### 3.1. Structural characterization by FTIR technique

**Figure 2:**
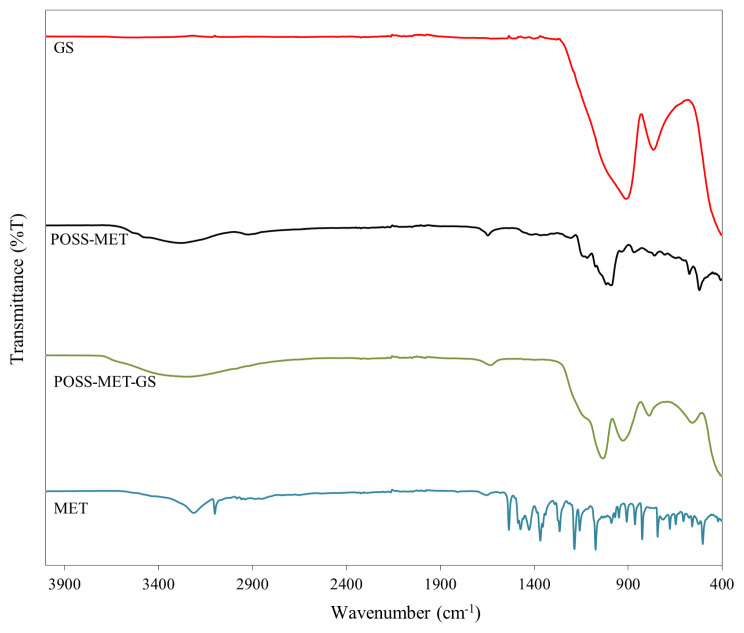
FTIR spectrum of the GS, POSS-MET, POSS-MET-GS and MET structures.

In Figure 2, we can see the FTIR spectra of POSS-based film structure containing metronidazole (POSS-MET), pure metronidazole (MET), glass coated with POSS-based film (POSS-MET-GS) and empty glass surfaces (GS). Metronidazole is a very small antibacterial diazole molecule. There is one nitro group and aliphatic methylene in the structure except for the diazo ring. It also carries one hydroxyl group. The asymmetric NO_2_ stretching vibration originating from the nitro groups in the structure is 1505 cm^−1^ and the symmetric NO_2_ stretching vibrations occur at 1360 cm^−1^. We see C-N stretching vibrations at 1442 cm^−1^ on the diazole ring and CH_2_ structured C-H peaks with aliphatic character at 925 cm^−1^. Again, the C-H stretching vibrations of the methylene group of the CH_3_ structure in the aliphatic character are at 2846–2941 cm^−1^ as a binary peak. In the study, pure metronidazole groups are linked via −OH linkage. In the FTIR spectrum of pure metronidazole structure, the −OH tensile vibration is observed at 3217 cm^−1^. Si-O-Si peak on the uncoated glass surface is clearly seen at 1100 cm^−1^. In the FTIR spectrum of POSS-metronidazole structure, we see the peaks arising from both POSS structure and metronidazole groups. Especially, we see a broad Si-O− bond band originating from the POSS structure at 1000–1100 cm^−1^ in the spectrum and symmetric stretching peaks for the Si-O-Si bond at 928 and 770 cm^−1^. In addition to, this structure there are C-C stretching peaks at 1092 cm^−1^, NO_2_ stretching peaks at approximately 1500 cm^−1,^ C-N tensile vibrations at approximately 1410 cm^−1^ and aliphatic C-H stretching peak at 2850–2950 cm^−1^ originating from metronidazole groups. In the FTIR spectrum of the coated surface, we see similar peaks. This proves to us that the coating is successful. FTIR spectral locations are consistent with the spectral results of previous studies [24, 25]. In the FTIR spectrum of the coated surface, two different Si-O-Si tensile peaks belonging to Si-O-Si groups, from POSS structure [26]. TEOS and glass surface gave the spectra at 1000–1100 cm^−1^ respectively as in previous studies [27, 28]. All these results prove that the desired coating is obtained.

### 3.2. Determination of structural and surface morphology properties by upright optic microscope, SEM-EDX and AFM techniques

**Figure 3:**
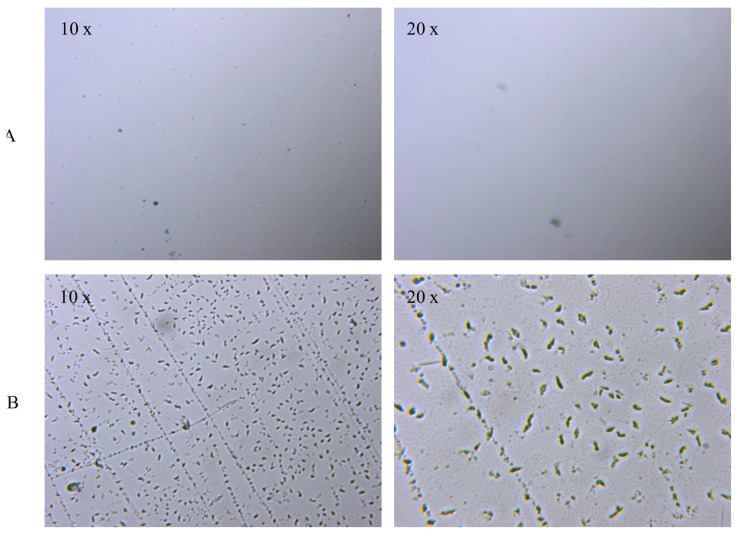
Optic microscope images of the GS (A) and POSS-MET-GS (B) structures.

**Figure 4:**
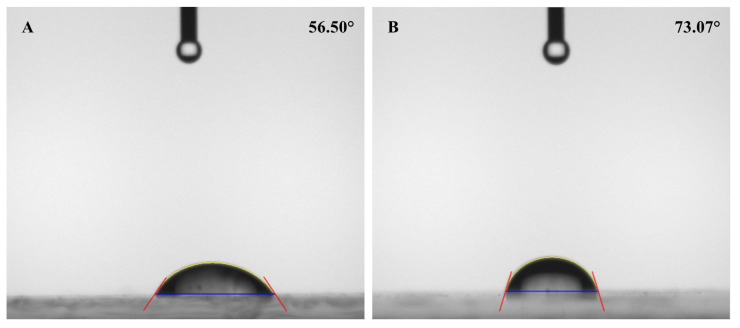
Contac angle images of the GS (A), and POSS based film coated glass surface (POSS-MET-GS) (B).

**Figure 5:**
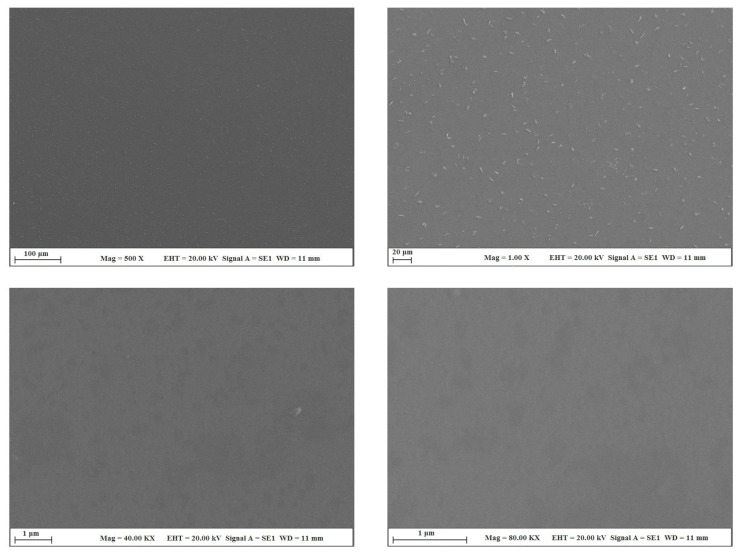
SEM images of the bare GS surface.

**Figure 6:**
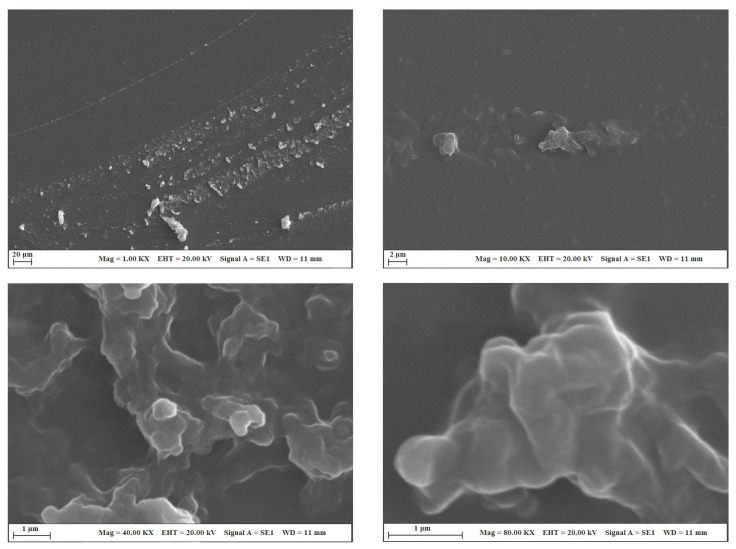
SEM images of the POSS-MET-GS coated glass surface.

Figure 3 shows the optical microscope images of the uncoated glass surface and the POSS-MET film coated surface. In the microscope images, given at 10× and 20× magnifications, the glass surface is smooth and homogeneous. However, POSS-MET film coated surface shows evenly distributed structures. These structures originate from metronidazole groups which are distributed between POSS and silica groups which are glassy in character. The particles dispersed on the coating surface show a uniform and homogeneous distribution. Thanks to these coatings on the glass surface, the hydrophilic character of the surface changes. In Figure 4, contact angle images and water contact angle values of the GS surface and POSS-MET film coated glass surface were given while the water contact angle value of the glass surface was 56.50° [29], the water contact angle of the POSS-MET film coated surface increased to 73.07°. This increase is due to aliphatic groups in POSS structure. In Figures 5 and 6, SEM images of GS surface and POSS-MET film coated glass surface are given at different magnifications. Especially the GS surface is smooth and glazed. SEM image of the bare glass surface is similar in the study by Li et al. [30]. However, the POSS-MET film coated glass surface has a cavity structure that is clearly visible at high magnifications. Surface roughness is due to metronidazole groups in the coating structure. At low magnifications and visually, the cavity structure on the surface of the coating is noticeably altered. Therefore, the coating does not significantly affect surface aesthetics.

**Figure 7:**
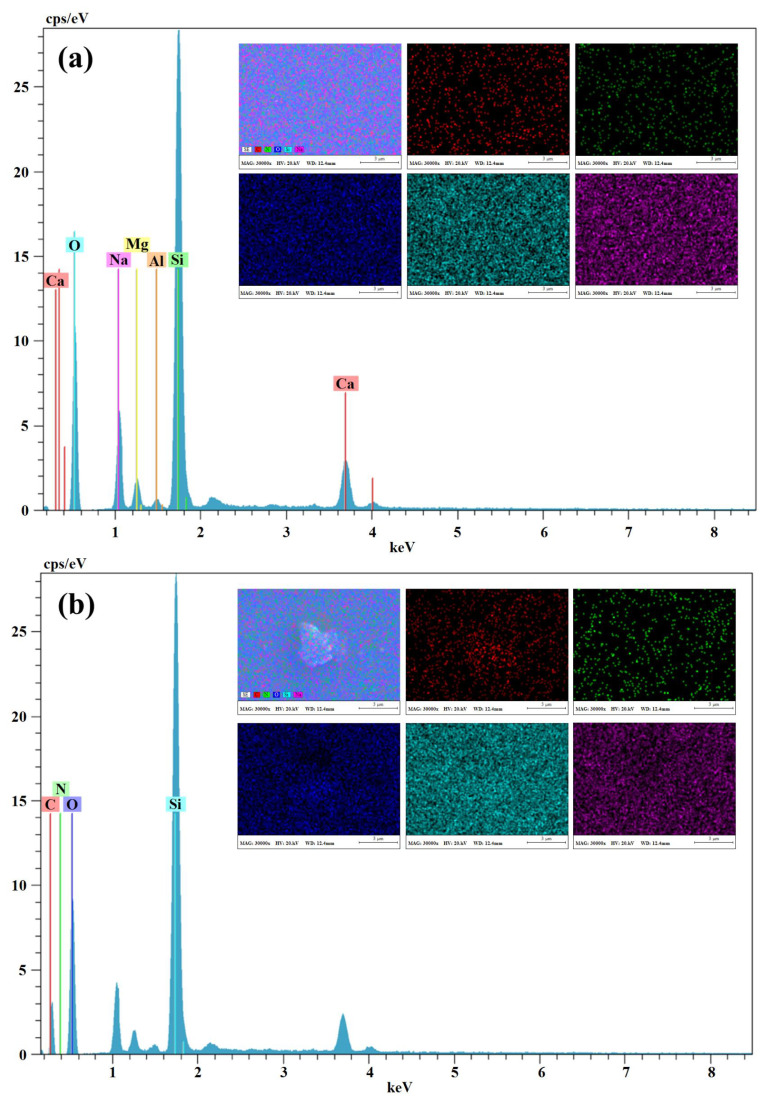
EDX spectrum of the GS (a), and POSS-MET-GS (b) coated glass surface.

**Figure 8:**
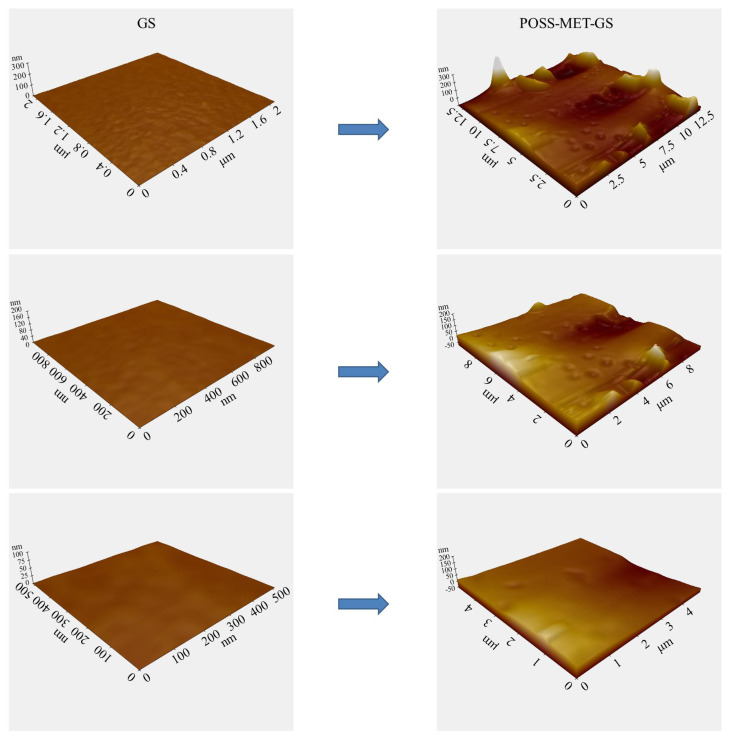
AFM images of the GS (left), and POSS-MET-GS coated glass surface.

**Figure 9:**
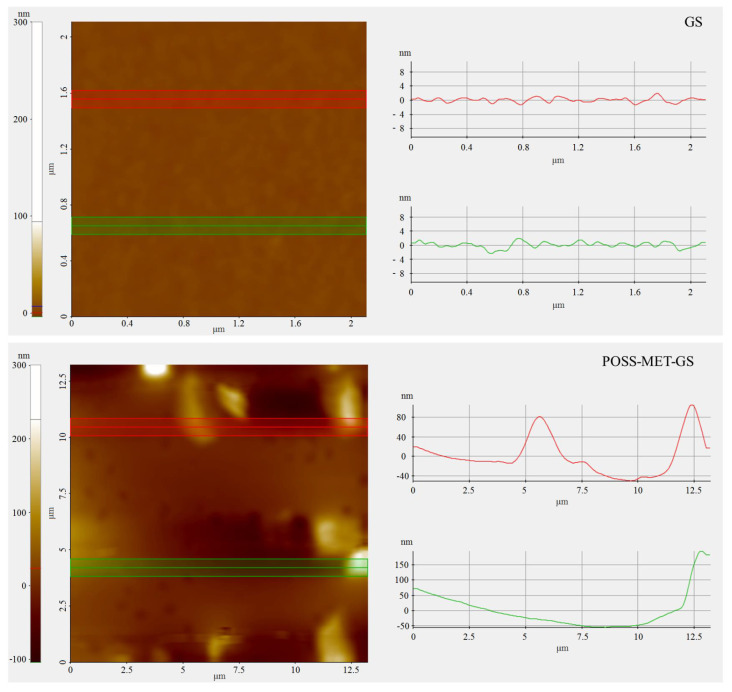
AFM topography and surface roughness of the GS, and POSS-MET-GS coated glass surface.

EDX analysis was performed to determine the change of elemental title on the surface of the coatings and EDX spectrum of the GS (a), and POSS-MET-GS (b) coated glass surface results are given in Figure 7. In these EDX spectrum results, peaks of uncoated GS structure Ca, Na, Mg, Al, Si and O atoms are seen. In addition, there are peaks of C, N, Ca, Na, Mg, Al, Si and O atoms in the EDX spectrum of the POSS-MET-GS structure. The peaks of the C and N atoms originate from the metronidazole structure and this molecule shows that it is homogeneously distributed in the structure. The results of this analysis prove that the desired coating structure is obtained homogeneously and purely on the GS surface. Film coating structure was also determined by AFM measurements. Figures 8 and 9 show AFM images of both uncoated glass surfaces and POSS-MET film coated glass surfaces. The uncoated glass surface AFM image is consistent with the bare glass surface image taken for different articles [31]. AFM images were taken at different magnifications on the uncoated glass surface (GS) and POSS-MET film coated glass surface. In these images, surface structure, morphology and roughness have changed significantly after coating.

### 3.3. Metronidazol release from POSS-based film coating structure

**Figure 10:**
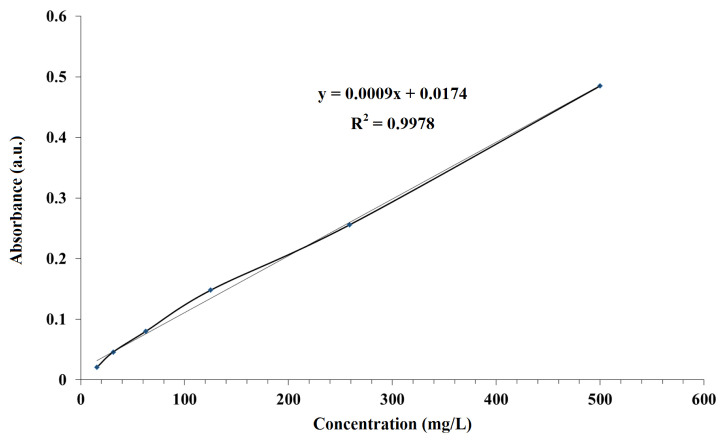
Standard calibration graph for metronidazole.

**Figure 11:**
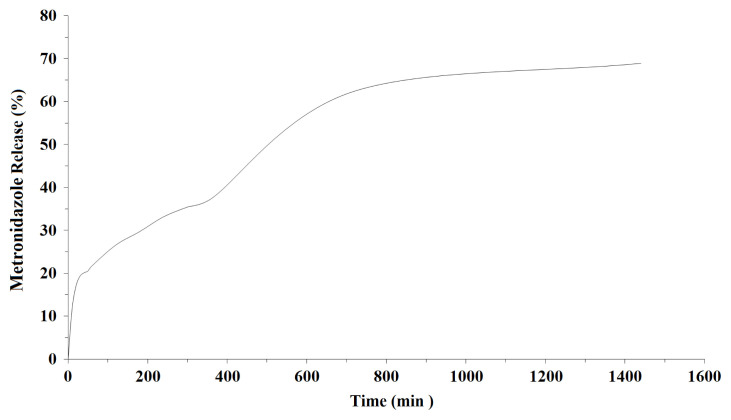
Metronidazole release (%) graph.

On glass surfaces coated with metronidazole containing POSS film, standard calibration graph for metronidazole was plotted before measuring with UV spectrophotometer (Figure 10). Standard curves were prepared for metronidazole in the PBS buffer (pH = 7.4) so that absorbencies could be converted to concentrations using the Lambert-Bear law [32]. Absorbance values at 340 nm wavelength between 10, 20, 30, 40, 50 min and 1, 2, 3, 4, 5, 6, 12 and 24 h were determined for metronidazole release from POSS-MET film coated glass surfaces. The released metronidazole amounts were calculated with measured absorbance values. The percentage metronidazole release versus time graph is given in Figure 11. When we look at the drug release graph, we see that the release begins in 10 min (11.66%) and increases slowly. The highest release occurs between 6 and 12 h. We see the maximum drug release as 68.90% in 24 h.

These results prove that metronidazole is bound to the synthesized film coating structure and that bound metronidazole is released and exhibits antibacterial activity.

### 3.4. Antibacterial activity results of POSS-MET film coated glass surface

**Figure 12:**
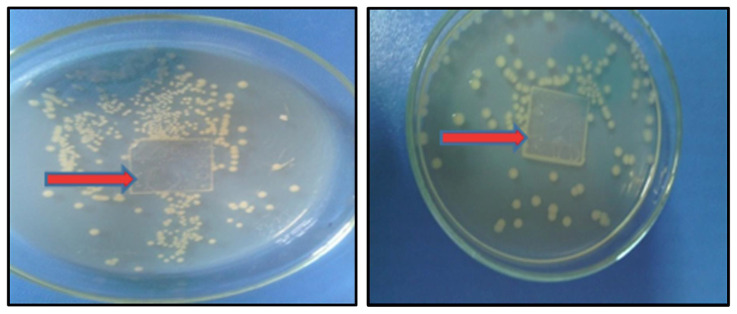
Images of glass surface (left) and POSS-MET film coated glass surface (right).

Antibacterial activity of POSS-MET film coated and uncoated glass surface were tested against *E.coli* and *S. aureus* bacteria. Results were given according to broth microdilution method, which determines the minimum inhibitory concentration (MIC) leading to the inhibition of bacterial growth. When we look at Figure 12, we see bacterial colonies under the uncoated glass surface, while we see that there are no bacterial colonies under the glass surface covered with POSS-MET film. The results are presented in Table.

In literature, most of the studies related to metronidazole are drug release studies. Metronidazole loaded pectin films and metronidazole loaded hydrogels drug delivery systems have been developed [33,34]. Metronidazole doped bacterial cellulose membranes are designed as cover material [35]. Metronidazole loaded polycaprolactone microspheres were prepared [36]. For the release of metronidazole, silica, silica/polydimethylsiloxane and silica/ polydimethylsiloxane/calcium cryogels were examined as polymeric carriers [37]. Nanoporous SiO_2_-CaO-P_2_0_5_ and HPC-SiO_2_-CaO-P_2_O_5_ xerogels were prepared for the release of metronidazole [38]. In this study, POSS-based antibacterial film synthesis was carried out, especially for biomedical surface coating areas.

## 4. Conclusion

In this study, POSS-based antibacterial film coating structure was successfully synthesized by the sol-gel method. In surface coating, POSS-based structures are encountered in many areas. Li et al. developed an amphiphilic, antifogging and anti-icing polyhedral oligomeric silsesquioxane-poly [2-(dimethylamino) -ethyl methacrylate]-block-poly(sulfabetaine methacrylate(POSS-PDMAEMA-b-PSBMA) coating [39]. Bakhshi et al. have developed a new nanocomposite polymer surface coating based on POSS and poly (carbonate-urea) to increase stent surface resistance [40]. Devaux et al. obtained polyurethane/POSS nanocomposite surfaces as a flame retardant coating for polyester and cotton fabrics [41]. When we look at the current studies, we see that the POSS-based film coating structures we synthesized offer an alternative contribution to the literature.

## Figures and Tables

**Table:** Minimum inhibition concentrations [MIC (μg/mL)] of POSS-MET film coating structure.

Sample	MIC (μg/mL)
Metronidazole containing POSS based film coating structure	*E. coli* = 568 *S. aureus* = 547
